# Distributional Variations in the Quantitative Cortical and Trabecular Bone Radiographic Measurements of Mandible, between Male and Female Populations of Korea, and its Utilization

**DOI:** 10.1371/journal.pone.0167992

**Published:** 2016-12-21

**Authors:** Muthu Subash Kavitha, Soon-Yong Park, Min-Suk Heo, Sung-Il Chien

**Affiliations:** 1 Department of Computer Vision and Image Processing, School of Electronics Engineering, Kyungpook National University, Daegu, South Korea; 2 Department of Computer and Robot Vision, School of Computer Science and Engineering, Kyungpook National University, Daegu, South Korea; 3 Department of Oral and Maxillofacial Radiology, School of Dentistry, Seoul National niversity, Seoul, South Korea; Medical University of South Carolina, UNITED STATES

## Abstract

It is important to investigate the irregularities in aging-associated changes in bone, between men and women for bone strength and osteoporosis. The purpose of this study was to characterize the changes and associations of mandibular cortical and trabecular bone measures of men and women based on age and to the evaluation of cortical shape categories, in a large Korean population. Panoramic radiographs of 1047 subjects (603 women and 444 men) aged between 15 to 90 years were used. Mandibular cortical width (MCW), mandibular cortical index (MCI), and fractal dimensions (FD) of the molar, premolar, and anterior regions of the mandibular trabecular bone were measured. Study subjects were grouped into six 10-years age groups. A local linear regression smoothing with bootstrap resampling for robust fitting of data was used to estimate the relationship between radiographic mandibular variables and age groups as well as genders. The mean age of women (49.56 ± 19.5 years) was significantly higher than that of men (45.57 ± 19.6 years). The MCW of men and women (3.17mm and 2.91mm, respectively, *p* < 0.0001) was strongly associated with age and MCI. Indeed, trabecular measures also correlated with age in men (*r >* −0.140, *p* = 0.003), though not as strongly as in women (*r >* −0.210, *p* < 0.0001). In men aged over 55 years, only MCW was significantly associated (*r* = −0.412, *p* < 0.0001). Furthermore, by comparison of mandibular variables from different age groups and MCI categories, the results suggest that MCW was detected to be strongly associated in both men and women for the detection of bone strength and osteoporosis. The FD measures revealed relatively higher association with age among women than men, but not as strong as MCW.

## Introduction

Osteoporosis is a huge personal and economic burden. The combined lifetime risk of hip, forearm, and vertebral fractures coming to clinical attention is around 40%, which is equivalent to the risk of cardiovascular diseases [1]. The high risk for a majority of individuals, who have already had at least one osteoporotic fracture, is neither identified nor treated [2]. In Korea, lifetime risk of osteoporosis-related fractures was estimated to be 59.5% and 23.8% for women and men aged over 50, respectively [3]. In addition, mortality rate after hip fractures was approximately 16% and 28% within 1 and 2 years, respectively [4]. Therefore, diagnosis at an early-stage may be important to detect and prevent osteoporotic fractures in men and women from different demographic groups.

Panoramic radiograph is an extensively utilized imaging modality in oral and maxillofacial surgical practices [5]. Furthermore, dental panoramic radiographs (DPRs) can be extended into the third dimension for obtaining more quantitative information of cortical and trabecular bone geometry based on x-ray physics transformation method [6]. Bone changes may be detected early on dental radiographs because bone formation rate is high during the mandibular alveolar process. [7, 8] Dental panoramic radiographic measurements, such as mandibular cortical index (MCI) or shape, mandibular cortical width (MCW) or mental index (MI), panoramic mandibular index (PMI), gonial angle, and fractal dimension (FD) of cortical as well as trabecular bone, have been applied to differentiate between individuals with or without osteoporosis. In fact, measurement of cortical thickness and FD has been demonstrated to be effective in analyzing bone microarchitecture of several skeletal sites [9–11]. Only a few studies have examined these measures on dental panoramic radiographs of men, with controversial predictions for the assessment of osteoporosis [12–14]. However, it was realized that the mandibular region has a significantly higher clinical measurement rate than the maxillary region [15].

The appropriate indicator in these radiographic measurements is MCW, and it has been largely studied for osteoporosis detection by measuring reduction in inferior mandibular cortex in women with low bone mineral density (BMD) or osteoporosis [16–18]. It could be useful in the detection of osteoporosis and hip fractures, because uniform bone loss was observed in the mandibular cortical bone and the hip [16]. The rapid reduction in the average cortical width values was observed in women aged between 50 and 70 years, compared to that in men of the same age group [17]. Likewise, it was found that the estimation of cortical bone is equally effective in men and women, for the detection of osteoporotic changes [13]. However, a recent study analyzed the diagnostic ability of mandibular bone and revealed that MCW was more effective in women than in men [12].

The estimation of mandibular bone FD is another commonly used indicator of bone structure analysis. There have been many studies on the use of FD of trabecular bone to identify women with low BMD or osteoporosis [10, 18, 19]. The FD values of trabecular bone were found to be more prominent in detecting osteoporotic changes in postmenopausal female Brazilian subjects [19]. However, another recent study on Brazilian male (49) and female (84) subjects demonstrated that FD values of the cortical bone are more accurate than those of the trabecular bone [13]. Several studies revealed the association between these radiographic measurements and mandibular cortical erosion for identifying undetected low BMD or osteoporosis [20, 14, 21]. However, a few studies reported that evaluation of mandibular cortical erosion was not suitable and that it did not correlate with either mandibular or maxillary BMD to distinguish between osteoporotic and normal individuals [15, 22]. In addition, there have been several studies that suggested that rate of bone loss increases with advancing age, which may differ among skeletal sites and between men and women [12, 14–17]. Although both men and women are affected, the bone loss was demonstrated to be more rapid in women than in men [16, 17].

In order to recognize age related changes in bone strength of individuals, it is essential to realize the ranges of values of cortical and trabecular bone geometry and their contribution in the population studied. Hence, this study aimed to measure MCW and FDs of molar, premolar, and anterior regions of the trabecular bone using digital panoramic radiographs (DPRs) of Korean male and female subjects, to analyze the variation of these variables and their association with different age groups, and also investigate the interactions of MCW and FD measures with the evaluation of MCI categories.

## Materials and Methods

### Subjects

This study selected 1047 subjects (444 males and 603 females), who visited Seoul National University Hospital, Seoul, Korea, for treatment of dental diseases from April 2011 to June 2013, and their digital DPRs were acquired. The average age of male subjects was 45 years and that of female subjects was 49 years (ranging from 15 to 95 years). The age and gender of each subject was acquired from hospital electronic medical records. Since this was a retrospective study the research was permitted without consent based on the regulation. The Institutional Review Board of Seoul National University Hospital approved the study protocol. Authors were not directly involved with the medical treatment of the participants. Furthermore, there were no demographic information were collected for the subjects involved in the study. All digital DPRs were collected using the same digital panoramic machine (OP-100, Imaging Instrumentarium, Tuusula, Finland) at 8–10 mA, 17.6s and 70kVp voltage using automatic exposure control. Images were stored in JPEG format with a matrix of 1976 × 976 pixels.

### Radiographic measurements of the mandible

**Fig 1:**
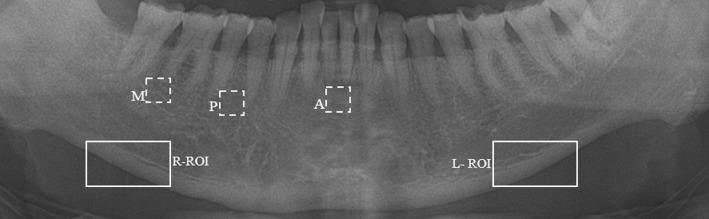
Digital panoramic radiograph with right (R) and left (L) regions of interest (ROI) for mandibular cortical width measurements and molar (M), premolar (P) and anterior (A) regions of trabecular bone for fractal dimension measurements.

MCW was determined from the panoramic radiographs; measured continuously to both right and left sides of the mandibular cortex at each point between the upper and lower boundaries of the cortical bone, similar to our previous study (Fig 1) [17]. Briefly, to differentiate cortical bone from trabecular bone and the background, pre-processing of the image was involved. Furthermore, to segment upper and lower cortical boundaries, the system calculates diameter and optimal path using distance transform and dynamic programming methods, respectively. The cortical boundaries were finally acquired as the envelope of the disc outlined by each pixel on the optimal path with its radius equalled the pixel value. After locating the cortical boundaries, the distance between these upper and lower boundaries was measured at each point along the line tangent to the polynomial curve using a second-order polynomial function. Mean MCW values from both left and right sides of the mandibular cortex were used in this study.

**Fig 2:**
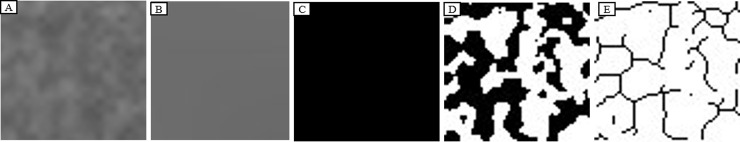
A. Original ROI cropped at molar region, B. Gaussian blurred image, C. Subtracted image, D. Thresholded image, E. Skeletonized image.

FD measures of the trabecular bone were estimated from three regions of interest (ROI) on the right side of the DPRs. The three ROIs (64 x 64 pixels) were molar, premolar, and anterior regions of the mandibular jaw bone. FD measures were calculated from all ROIs using ImageJ version 1.45s (National Institutes of Health, Bethesda, MD), as demonstrated previously [23]. Briefly, the duplicated image of the selected ROI was blurred using a Gaussian filter (sigma = 35). The resultant image, after subtracting the blurred image from the original image, was then converted to binary with threshold at a gray value of 128. It approximated the trabecular bone pattern on the segmented image. The binary image was then skeletonized, in which the skeletal and non-skeletal structures represented the trabecular bone pattern and bone marrow, respectively (Fig 2). The skeletonized image was utilized to estimate FD using box-counting method, in which the image was covered by a square grid of equally sized tiles. The widths of the square boxes applied to the image were 2, 3, 4, 6, 8, 12, 16, 32, and 64 pixels. Finally, FD was calculated from the slope of the line that fit the data points.

The mandibular cortical shape categories called MCI, were accessed by an oral radiologist (MSH), based on the appearance of the lower border of mandibular cortex distally from the mental foramen. The cortical shape was then categorized as normal cortex (C1, the endosteal margin of the cortex is even and sharp on both sides), mildly to moderately eroded cortex (C2, the endosteal margin shows semilunar defects or endosteal cortical residues), and severely eroded cortex (C3, the cortical layer forms heavy endosteal cortical residues and is clearly porous), according to the classification by Klemetti et al. [24].

### Statistical analyses

Subjects were categorized into six different age groups (15–24, 25–34, 35–44, 45–54, 55–64, and ≥65), for both genders. Mean and standard deviation were calculated for all radiographic mandibular variables used in this study. Normal distribution of the data was evaluated using the Shapiro-Wilk test. The relationships between radiographic mandibular variables and age as well as cortical shape categories of both genders were studied using Pearson correlation coefficients of linear regression model and one way analysis of variance. Tukey-Kramer test for pairwise comparisons (α = 0.05) was used to indicate significant differences in the interaction of estimated radiographic mandibular variables among age groups and cortical shape categories. The level of statistical significance was set to *p* < 0.05. Changes in variables between age groups were evaluated based on predicted values of a non-parametric function, local linear regression smoothing (loess), for robust fitting was used to identify differences between observed values for both genders [25]. Each regression was repeated using bootstrap resampling with 1000 iterations. The possibility of nonlinearities in age-related trends was examined by using polynomial terms in the models. The percentage difference in variables between age groups, as well as between genders, was estimated using loess, with gender as a factor variable. Differences between changes in variation in the variables and age as well as gender were tested using an age-sex interaction regression model. All statistical analyses were conducted using NCSS 11 statistical software (NCSS, Kaysville, UT) for windows.

## Results

Among the 1047 subjects, 603 (58%) were women and 444 (42%) were men. The mean and standard deviation of the radiographic mandibular variables of male and female subjects are presented in Table 1. Compared to men, women were significantly older (45.57 ± 19.6 years and 49.56 ± 19.5 years, respectively; *p* = 0.002) and had lower values of MCW (3.17 ± 0.28 mm and 2.91 ± 0.44 mm, respectively; *p* < 0.0001), FD at molar region (1.81 ± 0.11 and 1.77 ± 0.12, respectively; *p* < 0.0001), and FD at premolar region (1.81 ± 0.08 and 1.78 ± 0.09, respectively; *p* < 0.0001) for the detection of bone strength and osteoporosis. There was no significant difference in FD value at anterior region between men and women (men 1.78 ± 0.08, and women 1.79 ± 0.08; *p* = 0.468) observed in this study.

**Table 1:** Mean and standard deviation of the radiographic mandibular variables measured for men and women.

Variables	Men	Women	*P* value
MCW (in mm)	3.17 ± 0.28	2.91 ± 0.44	<0.0001***
FD_Molar	1.81 ± 0.11	1.77 ± 0.12	<0.0001***
FD_Premolar	1.81 ± 0.082	1.78 ± 0.097	<0.0001***
FD_Anterior	1.78 ± 0.084	1.79± 0.06	0.468

MCW, mandibular cortical width; FD, fractal dimension.

*** *P* < 0.0001

**Fig 3:**
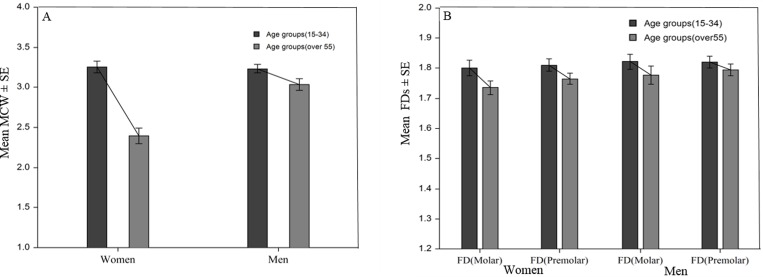
Age and gender associated mean cortical and trabecular variables of the mandible in men and women. Error bars represent standard errors and significance between each groups. (A) Mandibular cortical width (MCW). (B) Fractal dimensions (FD) of trabecular bone at molar and premolar regions.

The association of MCW and FD measures with different age groups and genders is given in Table 2. The correlation coefficients and *P*-values between radiographic mandibular variables and age among genders are presented in S1 Table. Age and gender were significantly associated with MCW. In women, MCW decreased rapidly, whereas in men it decreased slowly, after the mean age of 40 years. The FD measures at molar and premolar regions showed significant differences in men and women aged over 50 years. Compared to younger age groups (15–34), elderly age groups (above 55) of women had significantly lower MCW (3.23 ± 0.24 and 2.69 ± 0.51, respectively; *p* < 0.0001), FD at molar region (1.82 ± 0.11 and 1.76 ± 0.13, respectively; *p* < 0.0001), and FD at premolar region (1.81 ± 0.07 and 1.77 ± 0.10, respectively; *p* < 0.0001). Similarly among men, compared to younger age groups (15–34), elderly age groups (above 55) had significantly lower MCW (3.24 ± 0.20 and 3.06 ± 0.31, respectively; *p* < 0.0001), FD at molar region (1.83 ± 0.09 and 1.79 ± 0.13, respectively; *p* = 0.003), and FD at premolar region (1.82 ± 0.08 and 1.80 ± 0.08, respectively; *p* = 0.001) as described in Fig 3. Additionally, trabecular measures associated significantly with men, though not as strongly as with women is indicated in the overlapping standard error bar (Fig 3B). However, FD values at anterior region were not significantly different between younger and older age groups of both women and men. The correlation coefficients and *P*-values between MCW and age showed stronger negative correlation than FD measurements among genders are presented in S1 Table.

**Table 2:** Mean and standard deviation of the radiographic mandibular variables based on age and gender.

Parameters	Age groups (in years)					
	15–24	25–34	35–44	45–54	55–64	≥65
**Females**						
n (%)	84(13.9)	96(16.0)	51(8.5)	92(15.4)	136(22.6)	144(23.9)
Age (in years)	19.63 ± 2.96	28.99 ± 2.59***^a^	40.3 ± 2.91	50.10 ± 2.79***^c^	59.43 ± 2.99	73.83 ± 6.05***^b^
MCW (in mm)	3.25 ± 0.22	3.21 ± 0.26***^a^	3.10 ± 0.32	2.95 ± 0.36***^c^	2.79 ± 0.49	2.60 ± 0.52***^b^
FD_Molar	1.82 ± 0.09	1.81 ± 0.13**^a^	1.78 ± 0.11	1.77 ± 0.12	1.76 ± 0.13	1.75 ± 0.13***^b^
FD_Premolar	1.83 ± 0.07	1.79 ± 0.09**^a^	1.79 ± 0.09	1.77 ± 0.09	1.77 ± 0.09	1.76.10***^b^
FD_Anterior	1.80 ± 0.08	1.79 ± 0.08	1.79 ± 0.08	1.80 ± 0.07	1.80 ± 0.08	1.79 ± 0.09
**Males**						
n (%)	90(20.3)	72(16.2)	55(12.4)	66(14.9)	73(16.4)	88(19.8)
Age (in years)	20.37 ± 3.52	29.08 ± 2.90***^a^	40 ± 2.64	50.13 ± 2.79***^c^	59.12 ± 2.78	73.45 ± 5.89***^b^
MCW (in mm)	3.20 ± 0.22	3.28 ± 0.18	3.27 ± 0.19	3.20±0.31***^c^	3.12±0.31	2.99±0.31***^b^
FD_Molar	1.83 ± 0.09	1.82 ± 0.11	1.83±0.12	1.79±0.12	1.80±0.12	1.79±0.13 **^b^
FD_Premolar	1.82 ± 0.08	1.83 ± 0.07	1.82±0.07	1.82±0.08*^c^	1.80±0.09	1.80±0.07**^b^
FD_Anterior	1.78 ± 0.09	1.79 ± 0.07	1.76±0.10	1.79±0.09	1.79±0.06	1.78±0.08

n, number of subjects; MCW, mandibular cortical width; FD, fractal dimension.

* *P* < 0.05

** *P* < 0.005

*** *P* < 0.0001.

^a^ Comparison between age groups 15–34 and 35–54.

^b^ Comparison between age groups 15–34 and ≥55.

^c^ Comparison between age groups 35–54 and ≥55.

The ranges of MCW and FD measures in MCI categories are represented in Table 3. The correlation coefficients and *P*-values between radiographic mandibular variables and age groups of mandibular cortical index categories among genders are presented in S2 Table. Among men and women subjects, 79.0% and 53.0%, respectively, had a normal cortex, 17.0% and 33.0%, respectively, had a mildly or moderately eroded cortex, and 4.0% and 14.0%, respectively, had a severely eroded cortex. It was also found that subjects with severely eroded cortices (*p* < 0.0001) had a significantly greater mean age than of those with mildly or moderately eroded and normal cortices. Highly significant differences were found between MCW and MCI in both men and women. MCW was higher in C1 and C2 categories and lower in C3 category, in both genders. MCW among the three MCI categories of women was found to be significantly lower than that among the three categories of men. FD measures at molar and premolar regions were significant in women, but these measures were not significantly different in men among the three MCI categories. The correlation coefficients and *P*-values between radiographic mandibular variables and age of MCI categories showed stronger negative correlation in females than in males are presented in S2 Table.

**Table 3:** Mean and standard deviation of the radiographic mandibular variables based on mandibular cortical index and gender.

Parameters	C1	C2	C3
**Females**			
n (%)	321(53.0)	199 (33.0)	83(14.0)
Age (in years)	35.7 ± 14.4***^a^	63.28 ± 10.8**^c^	68.67 ± 10.8***^b^
MCW (in mm)	3.18 ± 0.21***^a^	2.78 ± 0.29***^c^	2.20 ± 0.45***^b^
FD_Molar	1.79 ± 0.11***^a^	1.75 ± 0.12*^c^	1.78 ± 0.10
FD_Premolar	1.79 ± 0.10*^a^	1.77 ± 0.10	1.78 ± 0.10
FD_Anterior	1.79 ± 0.06	1.79 ± 0.06	1.79 ± 0.07
**Males**			
n (%)	350 (79.0)	76 (17.0)	18 (4.0)
Age (in years)	40.52 ± 17.6***^a^	63.01 ± 14.3	68.91 ± 13.4***^b^
MCW (in mm)	3.24 ± 0.20***^a^	2.98 ± 0.29	2.55 ± 0.37***^b^
FD_Molar	1.81 ± 0.11	1.80 ± 0.11	1.78 ± 0.12
FD_Premolar	1.81 ± 0.08	1.80 ± 0.07	1.79 ± 0.08
FD_Anterior	1.78 ± 0.09	1.79 ± 0.08	1.78 ± 0.09

n, number of subjects; MCW, mandibular cortical width; FD, fractal dimension; C1, normal cortex; C2, mildly to moderately eroded cortex; C3, severely eroded cortex.

* *P* < 0.05

** *P* < 0.005

*** *P* < 0.0001.

^a^ Comparison between C1 and C2.

^b^ Comparison between C1 and C3.

^c^ Comparison between C2 and C3.

**Fig 4:**
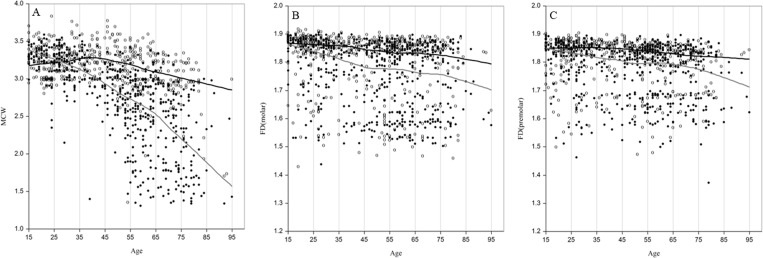
Age and gender specific changes in cortical and trabecular variables of the mandible in men and women. (A) Mandibular cortical width (MCW). (B) Fractal dimension (FD) of trabecular bone at molar region. (C) Fractal dimension of trabecular bone at premolar region. Curve fitting was done with a Loess smoother function. Individual values and smoother lines are given for men using open circles and solid lines (black) and for women using closed circles and solid lines (grey).

This study also investigated, in more detail, the age- and gender-specific changes in the mandibular variables by loess regression, namely a curvilinear representation of trend between the radiographic mandibular variables and age of men and women. A second degree polynomial line was fit at each calculation point with optimal smoothing parameter of 0.05 was used in the LOESS calculation. As demonstrated in Fig 4A, MCW was higher in men compared to women at every age and declined similarly in both genders. For the age group 35–44, women demonstrated a higher decline rate of MCW compared to men (3.4% and 0.4%, respectively; *p* < 0.0001). Similarly, for advancing age groups 45–54, 55–64, and ≥ 65, women demonstrated a higher decline rate of MCW, compared to men (women 4.6%, men 2.1%, *p* < 0.0001; women 5.0%, men 2.5%, *p* < 0.0001; and women 5.8%, men 3.9%, *p* < 0.0001; respectively). Thus in men, MCW decreased by 8.9% between the mean ages 40 and 73 years, whereas in women, MCW decreased by 18.8% between these ages. Hence, MCW decreased twice in women compared to men. The stable pattern of changes in FD measures at molar and premolar regions were quite similar in men and women (Fig 4B and 4C).

In women, FD at molar and premolar regions decreased by 3.3% and 1.9%, respectively, whereas in men, these measures decreased by 2.4% and 1.6%, respectively, between the mean ages of 40 and 73 years. Furthermore, the differences in changes in variations and predictive capability of these radiographic mandibular variables were examined and compared for all the subjects and elderly subjects (over 55 years), among both men and women (Table 4) for the detection of bone quality and osteoporosis. Of the total subjects, 36.0% men and 47.0% women were elderly subjects. MCW and FD measures showed highly significant negative correlation with all female subjects, whereas in males only MCW showed highly significant negative correlation and FD measures showed less significant associations, with all subjects. In all female subjects, MCW had strong significant association (*r* = −0.635, *p* < 0.0001) and FD values had low but significant association (*r >* −0.210, *p* < 0.0001), with age. In all male subjects, MCW had moderate significant relationship (*r* = −0.354, *p* < 0.0001) and FD values had poor but significant relationship (*r >* −0.140, *p* < 0.005), with age. For elderly male and female subjects, MCW showed moderately significant negative correlation (*r >* −0.410, *p* < 0.0001), whereas FD at molar (*r =* −0.076, *p* = 0.015) and premolar (*r =* −0.139, *p* = 0.019) regions showed significant poor negative correlation with women and no significant association with men. Hence, the predictive capability of variations in MCW with age and gender is significantly higher (*R*^2^ > 0.120, *p* < 0.0001) than that of variations in FD measures at molar and premolar regions for the assessment of bone quality.

**Table 4:** Comparison of radiographic mandibular variables between all and elderly male and female subjects.

Parameters	All subjects			Elderly subjects (aged over 55 years)		
**Females**	***R*^2^**	***r***	***p* value**	***R*^2^**	***r***	***p* value**
MCW (in mm)	0.403	−0.635	<0.0001	0.172	−0.414	<0.0001
FD_Molar	0.055	−0.235	<0.0001	0.018	−0.076	0.015
FD_Premolar	0.046	−0.214	<0.0001	0.019	−0.139	0.019
**Males**						
MCW (in mm)	0.125	−0.354	<0.0001	0.170	−0.412	<0.0001
FD_Molar	0.031	−0.171	0.001	0.010	−0.084	0.289
FD_Premolar	0.021	−0.142	0.003	0.001	−0.034	0.683

MCW, mandibular cortical width; FD, fractal dimension.

## Discussion

Qualitative and quantitative indices of panoramic radiographs have been developed by several studies for indication of bone resorption and osteoporosis [14, 15, 21, 22, 26]. To our knowledge, this is the first study to clarify aging-associated changes in MCW, and FDs for men and young men and women of Korean subjects. Hence, this study focused on measuring the radiographic mandibular variables and investigates the variation and association between these measurement variables and age groups as well as genders in a large study population. In addition, this study evaluated the associations of MCW and FD measures with the MCI categories. Results of this study suggest that MCW was significantly associated with age and MCI and could be a valuable measure for the assessment of bone strength and osteoporosis. Moreover, MCW and FDs declined similarly in men and women with advancing age; although, the decline rate of bone loss differed between the two sexes. Compared to women, men showed stronger bones and a higher resistance to fracture. FD measures at molar and premolar regions reveal a relatively better association between trabecular bone loss and age among women, than men (Table 4). FD at anterior region of the trabecular bone was not shown to be effective in men and women.

Although several studies have demonstrated the significance of mandibular cortical and trabecular bone morphologies in postmenopausal osteoporosis in elderly women, only a few studies have demonstrated these measurements in men [12, 13, 16]. A study by Roberts et al. demonstrated that MCW starts decreasing in women and men at ages 42.5 and 36 years, respectively [16]. Furthermore, it has been shown that the MCW reduction rate in females (13.4%) is much higher than in males (5.4%). Kalinowski et al recently reported that the inferior cortex width and MI have shown a steady decrease in bone loss after 30–39 years of age in a large Polish population [27]. In this study, the accelerated decrease in MCW with age was reflected relatively more in women (18.8%) than in men (8.9%), which were in agreement with previous studies with the observation of rapid decrease in women and slow decrease in men for the estimation of mandibular bone loss in osteoporosis [16, 27]. MCW of men increased from 10 to 30 years of age; however, in women, it did not show any increment after 20 years of age [17]. These findings were similar with the age and gender specific changes in MCW of men and women estimated in the current study (Table 2 and Fig 4A). Although these authors used different imaging techniques, regions of interest, and manual methods for measuring MCW, the loss of MCW with advancing age was double in women as compared to that in men, which is in agreement with other previous studies. Furthermore, this study confirms the strong association of MCW with both genders, supporting previous reports, among other radiographic mandibular variables estimated in this study (Table 4).

Several recent studies reported that the findings of cortical bone are more reliable than those of the trabecular bone for the diagnosis of osteoporosis [13, 28, 29]. For osteoporosis in Brazilian male and female subjects, MCW and FD analyses of mandibular cortical bone were found to be more reliable than that of anterior and inferior regions of the trabecular bone [13]. The qualitative and quantitative measurements using DPRs were significant only for MCW and FDs of the cortical bone; hence, cortical bone could be more suitable for diagnosis [28]. This is also true in study using other skeletal sites, which reported that the correlations of cortical measures are stronger than those of trabecular measures in both genders [30]. Strong association of MCW and weak association of FD measures with both genders, determined by correlations and squared R values by regression model (Table 4), was found in this study, which agrees with the aforementioned studies. In contrast to our results, the study by Alman et al. reported that FD of trabecular bone is a good indicator of low BMD in both men and women, whereas MCW is a poor indicator in men [12]. Furthermore, it was reported that MCW showed higher diagnostic ability as an indicator in older men than in younger men. However, Okabe et al. presented that the mandibular cortical findings in elderly subjects might be less beneficial for predicting fractures [31]. These differences in interactions and relationships between mandibular variables measured using DPRs could be due to differences in ROIs and in the composition and size of the subject population used for the evaluation. Moreover, there are differences in the methods used for cortical bone measurements in the aforementioned studies and in our study; hence, results of those studies might not be directly comparable to our results. FDs of the trabecular bone are widely used to identify the complexity and structure of the bone. It is calculated at various anatomical sites using different imaging modalities to observe the changes in bone pattern [29]. Watanabe at al reported that the FD of male group showed a higher degree of trabecular bone architecture and indicated that the structure of their bone is less prone to osteoporotic fractures [32]. This is similar to our study, with higher FD values for men at each age group, compared to women. Koh et al predicted that the lower premolar region was identified as the most proper site, among the mandibular and maxillary sites, for FD measurements, using a small number (56) of Korean population [26]. However, a single FD measured over the entire range may not be identified in the trabecular bone structure [33]. Although based on Korean population, the current study found mandibular molar and premolar regions of the trabecular bone was significant for the estimation of FD using DPRs. This is strongly supported by another study, which indicated that the relationship between age and bone mass at various sites of the mandible, and based on a large population over a greater age range, would be more robust in determining the appropriate site for measurement of bone loss [34].

In the determination of the radiographic appearance of the mandibular cortical bone based on male and female subjects, it was demonstrated that cortical thickness decreased with aging in women [14]. Furthermore, the validity of an MCI based on the cortical thickness was demonstrated as useful in screening for osteoporosis compared to BMD, which was also supported by the results of our study. The studies using cortical shape, detected from DPRs, to identify the skeletal status of women reported that MCI differed significantly with mandibular BMD, but it did not significantly differ with mandibular quantitative variables [22, 31]. However, in the current study highly significant differences were found between MCW and MCI for both genders. The majority of the aforementioned studies were performed measuring index with manual method. Furthermore, the lack of previous studies on the association of mandibular variables with MCI categories for both genders has made it difficult for us to compare our results directly. Moreover, most studies described potential indicators on radiographs using small sample populations of men and women [12, 13, 15, 17, 20, 21, 23, 26, 28, 29, 32], except a few studies that used larger sample size [14, 16, 27, 31], which limited comparison of results from this study. Although data on the subject’s age and gender were accessible, there were no other anthropomorphic data, such as height, weight, and medical data, available for the estimation of bone strength. Therefore, future studies should use clinical risk factors, such as exercise and use of medications, with the radiographic quantitative variables to diagnose osteoporotic fractures or bone strength. The mandibular trabecular bone was used for FD measurements in this study. However, the evaluation of FD of the mandibular cortical bone for various ages and genders and its relationship to FD of the trabecular bone would be important for generalizing the proper site for bone strength measurements.

In conclusion, gender analysis showed that MCW and FDs of trabecular bone were lower in women at every age, compared to men. MCW was strongly associated with age and MCI, in both men and women. The reduction rate of MCW was rapid and double in women, compared to men, whereas trabecular bone loss by FD measures were stable and quite similar in men and women. Regression model revealed that MCW could predict higher capability of variations with age and genders than the FD measures for the detection bone quality and osteoporosis. In addition, FD measures at molar and premolar regions revealed relatively better significance and correlation with age among women than men, but not as strong as MCW.

## Supporting Information

S1 Table Correlation coefficients and *P*-values between radiographic mandibular variables and age among genders. (DOC)

S2 Table Correlation coefficients and *P*-values between radiographic mandibular variables and age of mandibular cortical index categories among genders. (DOC)
