# Supplementary material for: Distributional Variations in the Quantitative Cortical and Trabecular Bone Radiographic Measurements of Mandible, between Male and Female Populations of Korea, and its Utilization
Source: PLoS One. 2016 Dec 21;11(12):e0167992. doi: 10.1371/journal.pone.0167992 (PMC5176279; doi:10.1371/journal.pone.0167992)
Supplement: S1 Table — (DOC) [file pone.0167992.s001.doc]

| **Correlation coefficient (*P*-value)** | | | | | | |
| --- | --- | --- | --- | --- | --- | --- |
| **Females** | 15-24 | 25-34 | 35-44 | 45-54 | 55-64 | ≥65 |
| MCW (mm) | -0.393 (.0002) | -0.360 (.0003) | -0.446 (.0001) | -0.339 (.009) | -0.009 (.916) | -0.487 (.0001) |
| FD_Molar | -0.22 (.042) | 0.019 (.850) | -0.151 (.291) | 0.159 (.131) | -0.054 (.533) | -0.102 (.222) |
| FD_Premolar | -0.211 (.054) | 0.109 (.289) | -0.131 (.360) | 0.179 (.089) | -0.097 (.264) | -0.245 (.003) |
| FD_Anterior | 0.146 (.186) | -0.269 (.008) | -0.163 (.255) | 0.131 (.213) | 0.012 (.888) | -0.152 (.069) |
| **Males** | 15-24 | 25-34 | 35-44 | 45-54 | 55-64 | ≥65 |
| MCW (mm) | -0.171 (.108) | 0.187 (.116) | 0.107 (.439) | -0.487 (.0001) | -0.192 (.104) | -0.563 (.0001) |
| FD_Molar | -0.049 (.642) | 0.155 (.193) | -0.070 (.611) | -0.275 (.025) | -0.076 (.521) | -0.104 (.337) |
| FD_Premolar | 0.055 (.607) | -0.113 (.345) | -0.003 (.986) | -0.315 (.010) | 0.075 (.529) | 0.079 (.460) |
| FD_Anterior | 0.023 (.833) | -0.084 (.483) | 0.051 (.714) | -0.067 (.593) | -0.158 (.181) | 0.056 (.603) |

**S1 Table**. **Correlation coefficients and *P*-values between radiographic mandibular variables and age among genders.**
