# Supplementary material for: Distributional Variations in the Quantitative Cortical and Trabecular Bone Radiographic Measurements of Mandible, between Male and Female Populations of Korea, and its Utilization
Source: PLoS One. 2016 Dec 21;11(12):e0167992. doi: 10.1371/journal.pone.0167992 (PMC5176279; doi:10.1371/journal.pone.0167992)
Supplement: S2 Table — (DOC) [file pone.0167992.s002.doc]

**S2 Table. Correlation coefficients and *P*-values between radiographic mandibular variables and age of mandibular cortical index categories among genders.**

| **Correlation coefficient (*P*-value)** | | | |
| --- | --- | --- | --- |
| **Females** | C1 | C2 | C3 |
| MCW (mm) | -0.408 (.0001) | -0.037 (.606) | -0.365(.0007) |
| FD_Molar | -0.267 (.0001) | -0.111 (.118) | -0.303(.005) |
| FD_Premolar | -0.235 (.0001) | -0.122 (.086) | -0.262(.017) |
| FD_Anterior | -0.034 (.546) | -0.098 (.169) | -0.222(.441) |
| **Males** | C1 | C2 | C3 |
| MCW (mm) | -0.069 (.197) | -0.247 (.029) | -0.453 (.059) |
| FD_Molar | -0.188 (.0004) | -0.207 (.069) | 0.209 (.405) |
| FD_Premolar | -0.113 (.034) | -0.272 (.016) | 0.177 (.482) |
| FD_Anterior | 0.006 (.909) | -0.034 (.769) | 0.076 (.762) |
